# *In vitro* fibrinolytic activity of an enzyme purified from Bacillus amyloliquefaciens strain KJ10 isolated from *soybean paste*

**DOI:** 10.1016/j.sjbs.2021.04.061

**Published:** 2021-05-01

**Authors:** Jayarajapazham Rajaselvam, Natarajan Benit, Saqer S. Alotaibi, M.A. Rathi, Srisesharam Srigopalram, Gurupatham Devadhasan Biji, Ponnuswamy Vijayaraghavan

**Affiliations:** a Bioprocess Engineering Division, Smykon Biotech Pvt LtD, Nagercoil, Kanyakumari, Tamil Nadu 629201, India; b Department of Botany, Holy Cross College, Nagercoil, Kanyakumari District, Tamil Nadu 629 001, India; c Department of Biotechnology, College of Science, Taif University, P.O. Box 11099, Taif 21944, Saudi Arabia; d Department of Biochemistry, Sree Narayana Guru College, Coimbatore, Tamil Nadu 641 105, India; e Application Specialist, Genurem Bisciences Tiruchirapalli, Tamil Nadu 620021,India; f Department of Zoology, Nesamony Memorial Christian College, Marthandam, Kanyakumari, Tamil Nadu 629 165, India

**Keywords:** *Bacillus amyloliquefaciens*, Fibrinolytic protease, Thrombolytic activity, *In vitro* clot lysis, Cardiovascular therapeutics

## Abstract

A fibrinolytic protease secreting producing Bacillus amyloliquefaciens strain KJ10 was initially screened from the fermented soybean. Maximum productivity was obtained in the culture medium after 40 h incubation, 34 °C incubation temperature at pH 8.0. Fibrinolytic protease production was enhanced in the culture medium with 1% sucrose (3712 ± 52 U/mL), 1% (w/v) yeast extract (3940 ± 28 U/mL) and 0.1% MgSO_4_ (3687 ± 38 U/mL). Enzyme was purified up to 22.9-fold with 26%recovery after Q-Sepharose HP column chromatography. After three steps purification, enzyme activity was 1606U/mg and SDS-PAGE analysis revealed 29 kDa protein and enzyme band was detected by zymograpy. Enzyme was highly active at pH 8.0, at wide temperature ranges (40 °C − 55 °C) and was activated by Mn^2+^ (102 ± 3.1%) and Mg^2+^ (101.4 ± 2.9%) ions. The purified fibrinolytic enzyme was highly specific against N-Suc-Ala-Ala-Pro-Phe-*p*NA (189 mmol/min/mL) and clot lytic activity reached 28 ± 1.8% within 60 min*in vitro*. The purified fibrinolytic enzyme showed least erythrocytic lysis activity confirmed safety to prevent various health risks, including hemolytic anemia. Based on this study, administration of fibrinolytic enzyme from B. amyloliquefaciens strain KJ10 is safe for clinical applications.

## 1 Introduction

Cardiovascular diseases, including stroke, high blood pressure, arrhythmias, peripheral vascular disease, valvular heart disease, are the leading causes of mortality in the world. Heart diseases are mainly responsible for about 29% mortality throughout the world (Mine et al., 2005). In pathophysiological condition stroke and myocardial infarction is the development of a blood clot, strongly binds to the blood vessels. In the blood clot, fibrin is an important component and is generally formed by the action of thrombin available in the fibrinogen. Accumulation of fibrin mainly enhances thrombosis in the circulatory system, mediating to cardiovascular diseases (Jeong et al., 2001). Plasminogen activator generated plasmin and hydrolyzed insoluble fibrin and fibrin degradation product is formed. Intravenous administration of plasminogenic activators generatesplasmin and hydrolyzed fibrin from the blood clot in the unbroken wall of the blood vessel. The administration of plasminogen activator from exogenous origin lysed blood clot and improved blood flow (Dobrovolsky and Titaeva, 2002). The fibrinolytic enzymes namely, streptokinase (EC 3.2.1.35), urokinase and tissue t-PA have been applied as the fibrinolytic agents (Mukhametova et al., 2002). These fibrinolytic enzymes are highly expensive, and cause various ill effects including, gastrointestinal bleeding (Blann et al., 2002, Biji et al., 2016). The efficacy and safety of fibrinolytic enzyme from microbial origin, especially from the genus *Bacillus* attracted more attention in recent years. Fibrinolytic proteases from microbial sources have good medical interest because of rapid growth, diversity and the production of enzyme by improved methods (Bajaj et al., 2013, Vijayaraghavan et al., 2016b). Other than *Bacillus* species, bacteria from the genus, *Pseudomonas* and *Staphylococcus* were reported for thrombolytic enzymes production (Simkhada et al., 2020). Enzyme cost is a significant deciding factor in clinical applications, and enzyme cost is minimized by using optimized medium (Taneja et al., 2017). In fermentation process, environmental-variables, nutrient-variables and ions potentially affect the growth and biosynthesis of enzymes and thus needs to be optimized before batch production (Singh and Bajaj, 2015, Vijayaraghavan et al., 2017). Purification and characterization of enzymes are useful to analyze the functional properties (Bizuye et al., 2014). In recent years, novel molecules have been isolated and characterized from various sources for several pharmaceutical and environmental applications (Theerthagiri et al., 2021, Ramu et al., 2020, Theerthagiri et al., 2019, Arasu et al., 2019). The present investigation was aimed on the analysis of novel fibrinolytic enzyme producing organism from the fermented food. The enzyme was assayed to analyze the influence of temperature, pH, enzyme kinetics and *in vitro* blood clot lytic ability.

## 2 Materials and methods

### 2.1 Screening of organism for the secretion of fibrinolytic enzymes

One gram sample was collected from the soybean paste (n = 6) for the screening of fibrinolytic protease secreting bacteria. The sample was serially diluted using physiological saline (0.9% NaCl) up to 10^-8^ dilution and plated. It was then kept for 24 h at 37 ± 1 °C in an incubator for the growth of bacteria. The isolated bacterial strain was further inoculated in the agar medium containing skimmed milk for the screening of protease production (primary screening). The individual culture showing clear zone on substrate plates were cultured on culture medium containing fibrin substrate (secondary screening). After 24 h growth of bacterium on fibrin plate, the zone of clearance was observed.

### 2.2 Characterization of fibrinolytic enzyme producing strain

The fibrinolytic enzyme producing organism was cultured and biochemical characters were analyzed. It was subjected for 16S rDNA gene sequence analysis. DNA was extracted using a commercial kit based on manufactures instructions (Merck, Germany). 16S rDNA gene was amplified using a forward (5ʹ- AGAGTRTGATCMTYGCTWAC-3ʹ) and a reverse (5ʹCGYTAMCTTWTTACGRCT-3ʹ) primer. The amplified 16S rDNA gene product sequence comparison was carried out with available database using BLAST analysis in NCBI server (Altschul et al., 1997).

### 2.3 Optimization of media components and fermentation conditions

Enzyme production was performed in the medium containing (%, w/v) dextrose – 1, peptone − 0.5, yeast extract − 0.5, MgSO_4_·7H_2_O − 0.02, K_2_HPO_4_ − 0.1, and KH_2_PO_4_ − 0.1(pH 7.2). The newly cultured bacterial strain in nutrient broth (18 h at 37 ± 1 °C) with Absorbance _600_ 0.840 ± 0.05 was used as seed culture (inoculums). The influence of fermentation time on fibrinolytic enzyme synthesis was obtained by performing the bacterial culture to various incubation times (12 h, 24 h, 36 h, 48 h, 60 h, 72 h, 84 h and 96 h). The culture was inoculated and incubated at various temperature ranges (25, 28, 31, 34, 37 and 41 °C) to determine optimum temperature. The pH of the production medium was maintained between 6.5 and 8.5. The influence of various carbon (1%, w/v) (starch, , sucrose, fructose, maltose, glucose, and xylose) and nitrogen (1%, w/v) (peptone, yeast extract, oat meal, beef extract, ammonium sulphate, and urea) and mineral supplements (0.1%, w/v) (NaH_2_PO_4_, KH_2_PO_4_, K_2_HPO_4_, MgSO_4_, and Na_2_HPO_4_) were evaluated. The tested minerals were prepared at 1% concentrations and supplemented with the production medium.

### 2.4 Purification of fibrinolytic enzyme

The fermented medium (150 mL) was centrifuged (10,000 × g, 10 min) and concentrated by ammonium sulphate (70%) and stored at 4 °C for 12 h. It was centrifuged at 10,000 × g for 10 min and dissolved in buffer A (sodium phosphate buffer, pH 7.2, 50 mM). The sample was dialyzed using a 15-KDa MWCO membrane for overnight against buffer A. After the completion of dialysis, the crude sample preparation was loaded on Q-Sepharose HP column (2.0 × 8 cm) which was pre equilibrated. Five column volume of buffer A was used to collect the proteins from the column using sodium chloride gradient (0 – 1 M). The volume of the fraction was 2.5 mL and the elution rate was 2.0 mL/min. The protein fractions were monitored and enzyme activity was tested. The enzyme fraction was concentrated to 3.0 mL and loaded on pre equilibrated Q-Sepharose HP chromatography column with buffer A (0.9 × 75 cm). The eluted enzyme fraction was lyophilized (Xin et al., 2019). SDS-PAGE was prepared using 12% separating gel and 5.5% stacking gel (Wasko et al., 2012). The protein of enzyme was assayed as suggested by Smith et al. (1985) and enzyme activity was teste using fibrin-agarose plate method (pH 7.2) (Astrup and Mullertz, 1952). Fibrinolytic enzyme activity was located in SDS-PAGE using fibrin substrate without any enzyme denaturation and heat treatment (Kim et al., 1998).

### 2.5 Properties of fibrinolytic enzyme

The impact of temperature and pH was evaluated as described by Hua et al. (2008). Optimum pH of enzyme was determined by assaying the enzyme over pH ranges of 4.0 – 10.0. The optimum temperature of enzyme was evaluated by assaying activity over wide temperature ranges of 25 – 70 °C. The residual enzyme activity was evaluated by analyzing the enzyme in buffers at various pH ranges (4.0 – 10). It was incubated at 40 °C for 1 h to evaluate the stability of enzyme on various buffers. To determine residual enzyme activity, sample was incubated at pH 8.0 with various temperature ranges (30 – 55 °C). The remaining activity of the sample was tested using buffer A containing 5 mM ions ((Ba^2+^, Zn^2+^,Ca^2+^, Mn^2+^, Mn^2+^, and Mg^2+^) (Hua et al., 2008). To analyze the kinetic constants, V_max_, and K_m_ was calculated using Lineweaver and Burk plot. To analyze substrate specificity, B. amyloliquefaciens strain KJ10 enzyme was incubated with synthetic substrates. The substrates used were, D-Val-leu-Arg-*p*N and N-Suc-Ala-Ala-Pro-Phe-*p*NA, for the determination kallilrein and chymotrypsin or subtilisin, respectively. The enzyme reaction contained synthetic substrate (1 mM), 2 U enzyme at pH 7.4 and experiment was done for 15 min and incubated at 40 °C. The released *p*NA was measured at 405 nm using analyzed and amidolytic activity was calculated.

### 2.6 Blood clot lytic property *in vitro* and haemolytic properties of fibrinolytic enzyme

To analyze the *in vitro* clot lytic activity, human blood (250 µL) was placed in a plastic sterile vial. To this CaCl_2_ (10 mM) was added in the tube. It was placed on water bath for 30 min (37 °C). After the formation of blood clot, enzyme was added at various concentrations (10 µL to 100 µL) and was kept at 37 °C for 5 h. After 5 h, clot lytic property was determined and the percentage blood clot lytic property was recorded (Holmstrom, 1965). Hemolytic property of fibrinolytic enzyme was assayed as suggested previously by measuring the the sample at 540 nm (Amin and Dannenfelser, 2006). Fibrinolytic enzyme was added at various doses (10 U, 20 U, 30 U, 40 U and 50 U). Millipore water and physiological saline (0.9% sodium chloride) were used as controls.

### 2.7 Data analysis

The values express the mean ± SD of triplicate individual works. One way analysis was performed and significance level (5%) was determined. The statistical software (STATISTICA, Version 5.5, Tulsa, USA) was applied for data processing.

## 3 Results and discussion

### 3.1 Isolation of fibrinolytic enzyme producing organism from the fermented food

Fibrinolytic enzymes from bacteria are useful to develop thrombolytic agent for the treatment of CVDs (Kotb, 2013). Microbial fibrinolytic enzymes degrade fibrin blood clot without any side effects. Fibrinolytic enzyme producing organism has been reported from various fermented food used in Asian countries. These include, *Bacillus amyloliquefaciens* LSSE-62 (Wei et al., 2012), and *Bacillus megaterium* KSK-07 (Kotb, 2015). A total of 21 different bacterial colonies were obtained from the fermentedsoybean paste and 17 bacterial isolates were selected. Protease activity was primarily screened for these 17 bacterial strains and only 11 isolates showed considerable activity (>10 mm zone of clearance). These 11 strains were tested for fibrinolytic activity on fibrin - agarose plates. Among the 11 isolates, the strain KJ10 showed 18 mm zone. The potent enzyme producing strain KJ10 was characterized based on biochemical properties and 16S rDNA sequencing. It was Gram-positive and utilized lactose. The bacterial colony showed white colouration, endospore forming, creamy, catalase positive, irregular shaped, motile, grown up to 45 °C in liquid culture. The strain KJ10 was identified as Bacillus amyloliquefaciens strain KJ10. Bacillus species produced various intracellular and extracellular proteases. These proteases degraded various natural proteins including fibrin substrate. These varied in properties namely, fibrin degradation pattern, nature, molecular weight and specificity. Fibrinolytic enzymes from food grade organisms can be used for thrombosis related diseases/disorders (Peng et al., 2005, Kim et al., 1996, Hu et al., 2019). The screened bacterial strain KJ10 was Gram’s positive and motile. The biochemical properties were described in Table 1.

**Table 1:** Biochemical and physiological characters of bacterial stain.

Gram's staining	+
Catalase test	+
Methyl red	+
Citrate test	+
Nitrite reduction	+
Nitrate reduction	+
Casein hydrolysis	+
Lipase production	+
Glucose fermentation	+
Xylitol	–
Mannitol	+
Maltose	+
Lactose	+
D-galactose	–
Arabitol	+
Cellobiose	–

+positive reaction; − negative reaction.

### 3.2 Effect of environmental sources on fibrinolytic enzymes production

**Fig 1:**
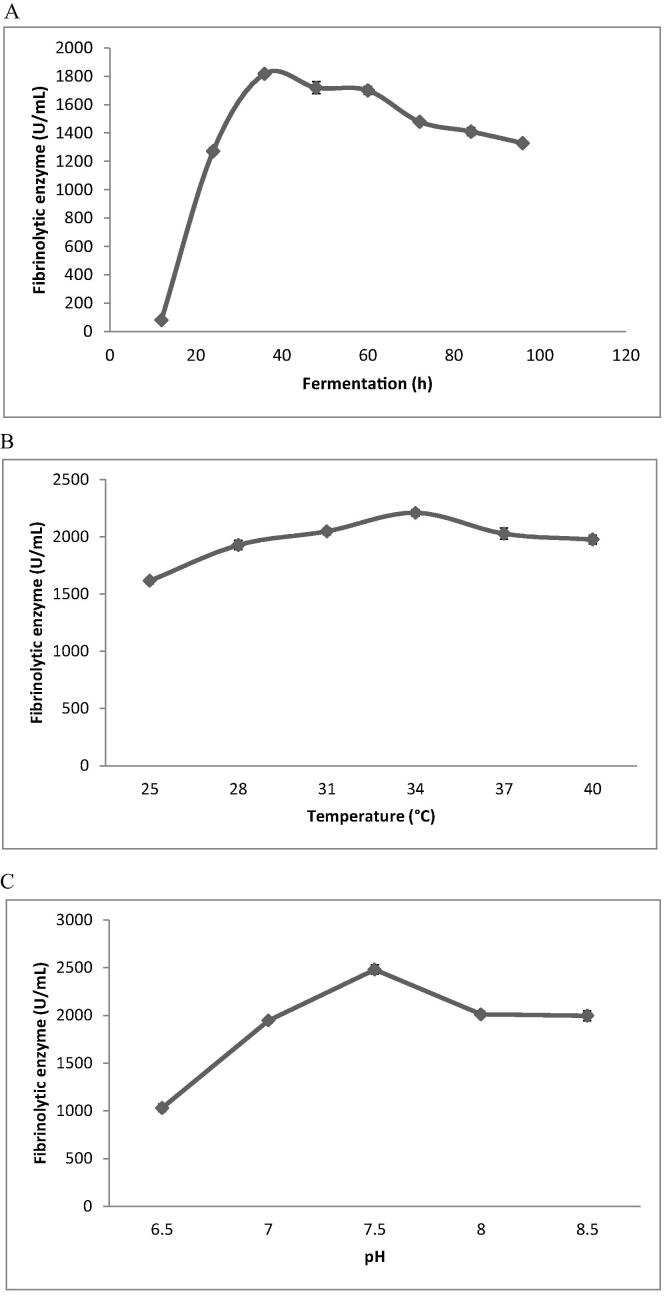
Production of fibrinolytic enzymes in optimized condition (A-C). Effect of incubation period (A), temperature (B) and effect of pH (C).

The strain KJ10 produced maximum amount of fibrinolyitc enzyme after 40 h incubation (Fig. 1A) and declined gradually. Enzyme production is mainly related to the nutrient components, nature of the medium and bacteria. In *Bacillus subtilis* Egy, fibrinolytic protease productivity was high after 96 h (Moharam et al., 2019). Enzyme production in relation with incubation temperature was analyzed. Fibrinolytic enzyme production increased gradually and attained maximum at 34 °C (2209 ± 29 U/mL) and slowly declined at higher temperature (Fig. 1B). This result was highly agreed with *B. subtilis* subsp. natto (Cho et al., 2010). The findings presented in Fig. 1C showed that pH 8.0 is highly favoured for the production of fibrinolytic enzymes. *B. amyloliquefaciens*has been showed enhanced production of enzymes in near to neutral pH value or at alkaline condition. The pH of the culture medium influence on the growth and accumulation of various metabolites from the medium required for maintaining the stability and maintain function and structure of the enzyme (Zheng et al., 2005).

**Fig 2:**
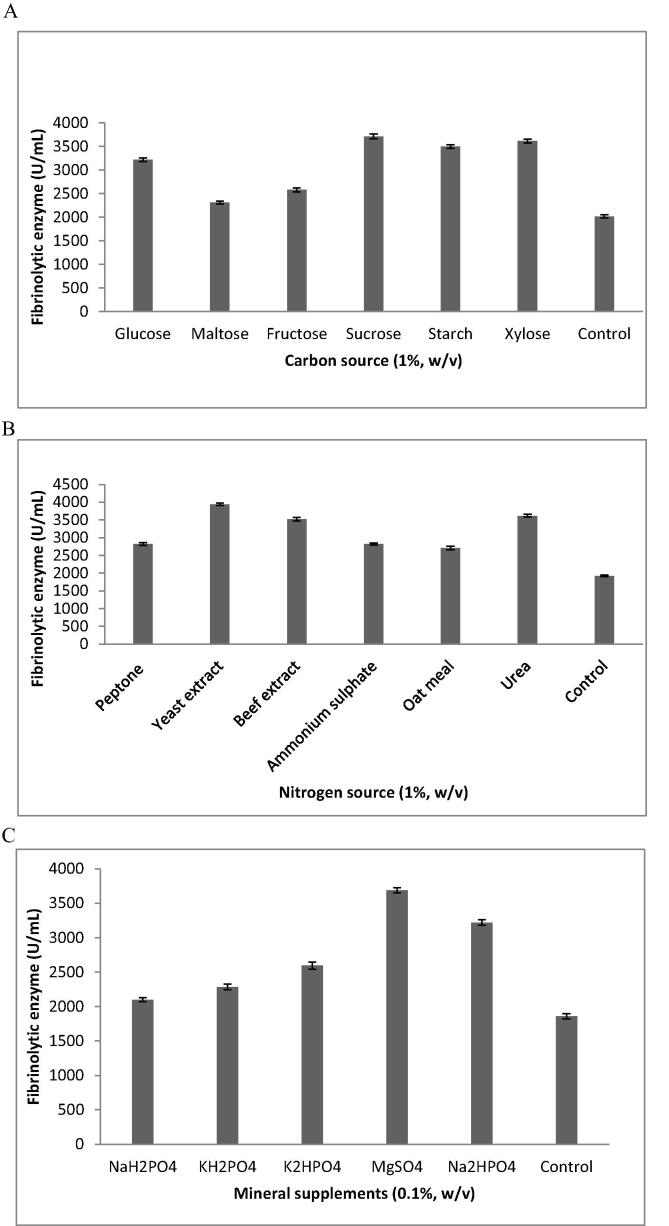
Effect of nutrient sources on fibrinolytic enzyme production from *B. amyloliquefaciens* KJ10. Culture was inoculated with production medium containing carbon sources (A), nitrogen sources (B) and mineral supplements (C) and incubated for 72 h.

### 3.3 Influence of nutrients on fibrinolytic enzymes production

**Fig 3:**
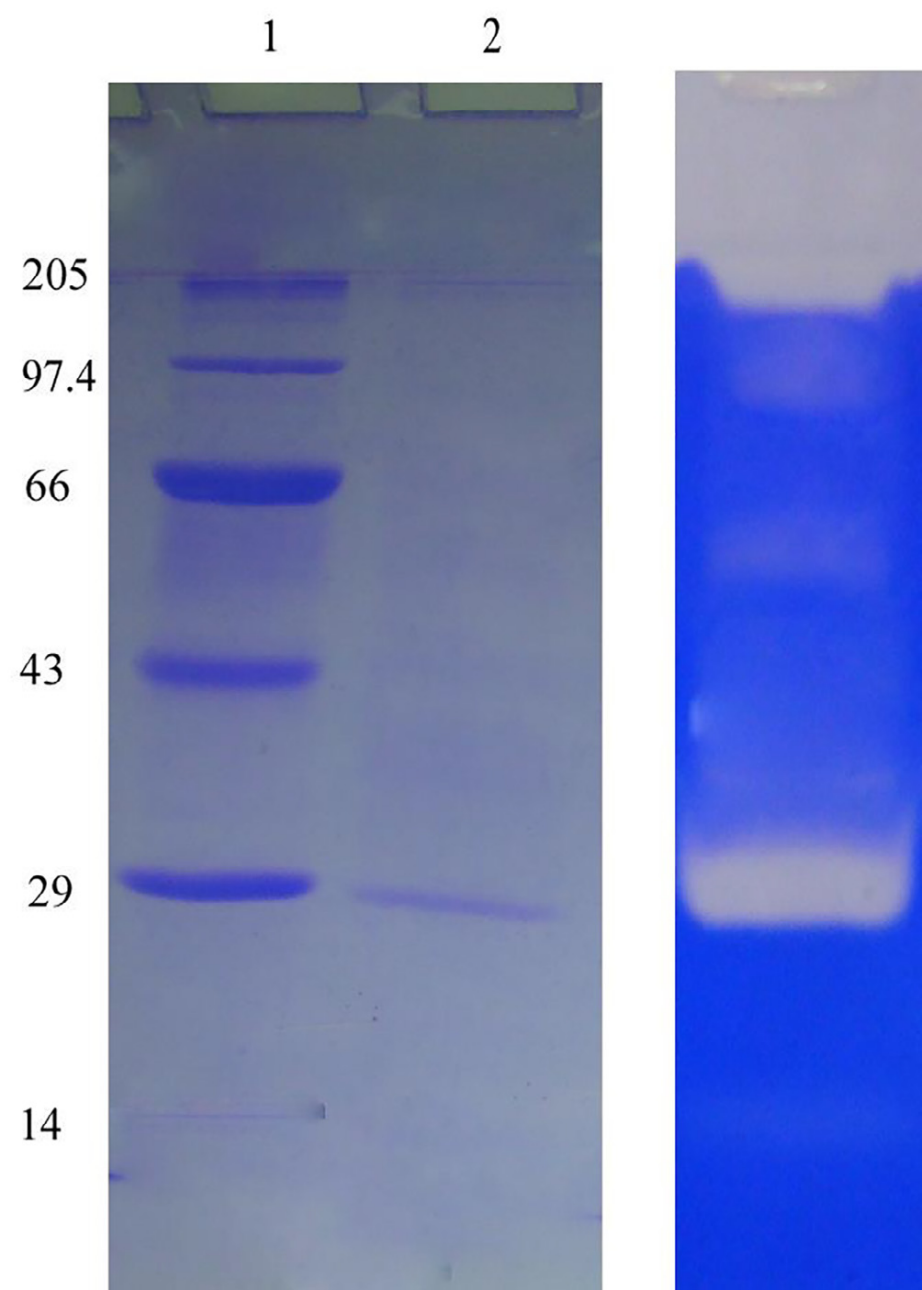
SDS-PAGE analysis of fibrinolytic enzyme from B. amyloliquefaciens strain KJ10. The purified fibrinolytic enzyme appeared as a single band corresponding to 29 kDa and the fibrinolytic enzyme appeared as a colourless band in zymography.

Carbon source has potential influence as described in Fig. 3 Fig. 2A, the strain *B. amyloliquefaciens* produced minimum quantity of enzyme (1030 U/mL) in the control experiment. In the production medium containing sucrose significantly enhanced fibrinolytic activity, which was significantly higher than that obtained using glucose, maltose, fructose, starch and xylose. Soluble starch showed less enzymes activity than other carbon sources (p < 0.05). Similar findings were achieved in the case of *Bacillus* sp. IND12, where sucrose has been used as the optimal carbon source for the production of fibrinolyic enzymes.As described in Fig 2B, the strain *B. amyloliquefaciens*produced high amount of enzyme (3940 ± 28 U/mL) (p < 0.05) in the yeast extract medium. Mineral salts are widely used to stimulate the production ofvarious biomolecules and enzymes. As described in Fig. 2C, supplemented MgSO_4_ (3687 ± 38 U/mL), Na_2_HPO_4_ (3219 ± 40 U/mL) and K_2_HPO_4_ (2596 ± 52 U/mL) increased enzyme production. The present finding was comparable with previous results. In *B. subtilis* P13, supplemented Mg^2+^ ions enhanced enzymes production (Pillai et al., 2011) and was maximum than other mineral salts.

### 3.4 Purification of fibrinolytic enzymes

Chromatography methods and salting out have widely used in the purification of various therapeutic enzymes (Mahajan et al., 2012). In this study, *B. amyloliquefaciens* proteolytic enzyme was purified by 22.9-fold than crude sample and the recovery rate was 26% (Table 2). *B. amyloliquefaciens* produced fibrinolytic proteases with molecular weight 29 kDa (Fig. 3). Mahajan et al. (2012) previously purified fibrinolytic enzyme from Bacillus strains with 7.5% enzyme recovery with 34.42-fold enzyme purification. In as study, Hu et al. (2019) characterized fibrinolytic protease from the genus *Bacillus* with 29 kDa after UNOsphere Q column chromatography, gel filtration chromatography and UNOsphere Q column chromatography.

**Table 2:** Purification summary of fibrinolytic protease from B. amyloliquefaciens strain KJ10.

Procedure	Total activity (Units)	Total protein (mg)	Specific activity (U/mg protein)	Purification (fold)	Yield (%)
Crude sample	14,212	203	70	1	100
Ammonium					
sulphate	11,410	69.2	164	2.34	80.2
Q-Sepharose HP	3694	2.3	1606	22.9	26

### 3.5 Characterization of enzyme

**Fig 4:**
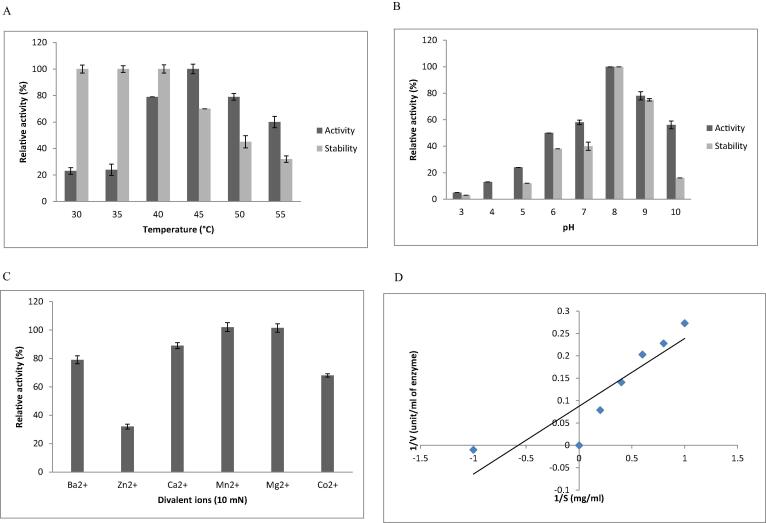
Properties of fibrinolytic enzyme from B. amyloliquefaciens strain KJ10. Effect of temperature activity and stability (A), pH on enzyme activity and stability (B) and ions enzyme stability (C), and enzyme kinetics analysis using Lineweaver and Burk plot (D).

The isolated fibrinolytic protease showed high activity and stability at 45 and 40 °C (Fig. 4A). This temperature optimum was higher than fibrinolytic enzyme from other proteases (Bajaj et al., 2013, Vijayaraghavan et al., 2016a). In the case of Streptomyces sp. CS624 and B. subtilis A26, temperature optimum was 60 °C (Mander et al., 2011, Agrebi et al., 2009). Vishalakshi et al. (2009) reported optimum activity of fibrinolytic protease of S. gulbargensis at 45 °C. Also enzyme was highly stable between 30 °C and 50 °C incubation temperature for 1 hwhich revealed efficient uses of this fibrinolytic enzyme in clinical applications. Enzyme activity was high at pH 8.0 (7.0 to 9.0) (Fig. 4B) which was similar with the analysis reported by Bajaj et al., 2013, Mahajan et al., 2012, Agrebi et al., 2010 in protease producing bacterial species. In the case of other *Bacillus* strains, enzyme activity with broad pH range was reported (Agrebi et al., 2009, Agrebi et al., 2010). The present finding also revealed high activity at broad pH ranges. Divalent ions effectively affect the property of fibrinolytic enzyme in various ways. *B. amyloliquefaciens* protease showed strong inhibition in the presence of Zn^2+^ (32 ± 1.7%), Co^2+^ (68 ± 1.2%) and Ba^2^ (79 ± 2.8%). However, *B. amyloliquefaciens* fibrinolytic enzyme was activated by Mn^2+^ (102 ± 3.1%) and Mg^2+^ (101.4 ± 2.9%) (Fig. 4C). In B. cereus NS-2, supplemented Mg^2+^ and Hg^2+^improved activity but inhibitory effort was reported by Fe^2+^ (Bajaj et al., 2013). B. subtilis A26 showed little influence of ions such as Mn^2+^, Mg^2+^, Cu^2^, Ba^2+^, K^+^ and Na^+^ (Agrebi et al., 2009, Vijayaraghavan et al., 2019). Enzyme kinetics was studied by reacting fibrin substrate with purified enzyme at various concentrations. The important kinetic factors such as, Km and Vmax of fibrinolytic enzyme was determined (Fig. 4D). The purified fibrinolytic enzyme was highly specific against N-Suc-Ala-Ala-Pro-Phe-*p*NA than D-Val-leu-Arg-*p*N. The substrate hydrolysis rate was 189 mmol/min/mL in N-Suc-Ala-Ala-Pro-Phe-*p*NA and it was low (148 mmol/min/mL) in D-Val-leu-Arg-*p*N (kallikrein).

### 3.6 *In vitro* blood clot lytic and haemolytic properties

**Fig 5:**
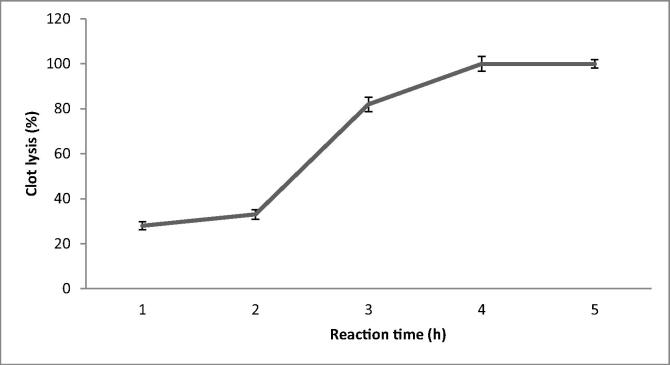
*In vitro* blood clot lytic activity of isolated fibrinolytic enzyme at various reaction times.

The fibrin blood clot lytic activity was determined *in vitro* and was 28 ± 1.8% after 1 h reaction. Clot lytic activity reached maximum (100%) after 4 h incubation (Fig. 5). At this reaction time, positive controls showed 31 ± 1.3% and 0% blood lytic activities. Fibrinolytic enzyme from *Bacillus subtilis* LD-8547 showed only 38.4% thrombolytic activity (Yuan et al., 2012). Cintra et al. (2012) used fibrinolytic enzyme from snake venom and reported 100% clot lytic activity within 24 h of reaction. Prasad et al. (2006) used streptokinase and revealed clot lytic activity ranged between 62.2 and 70.8% after 45 min of incubation. Fibrinolytic enzyme extracted from *Codium fragile* also showed 80% blood clot lytic activity (Choi et al., 2013). The purified fibrinolytic enzyme showed<3% erythrocytic lysis activity, which was similar to *Bacillus subtilis* LD-8547, which showed about 5% haemolytic property (Yuan et al., 2012). Amin and Dannenfelser (2006) reported more than 8.0% haemolytic activity *in vitro*. Analysis of haemolytic property is very important to prevent various health risks, including hemolytic anemia. Based on this study, administration of fibrinolytic enzyme from B. amyloliquefaciens strain KJ10 is safe for clinical applications.

## 4 Conclusions

In this research, a novel blood clot lytic enzyme secreting strain KJ10 was screened and it was characterized. This strain showed high productivity under optimized conditions in liquid culture. The development of optimized media is useful to enhance enzyme secretion. It completely lysed fibrin blood clot *in vitro* and less (<2.5%) haemolytic activity was determined. The characterized fibrinolytic enzyme is safe and has the potential to treat thrombosis.

## 5 Compliance with ethical standards**a

No experimental animals used for this study.

## Declaration of Competing Interest

The authors declare that they have no known competing financial interests or personal relationships that could have appeared to influence the work reported in this paper.
